# Organization of the Cytoskeleton in Ectopic Foci of the Endometrium with Rare Localization

**DOI:** 10.3390/biomedicines9080998

**Published:** 2021-08-11

**Authors:** Konstantin A. Toniyan, Victoria V. Povorova, Elena Yu. Gorbacheva, Valery V. Boyarintsev, Irina V. Ogneva

**Affiliations:** 1 Gynecology Department, FGBU KB1 (Volynskaya) UDP RF, 121352 Moscow, Russia; ktoniyan@mail.ru (K.A.T.); vkartveli13@yandex.ru (V.V.P.); Elenagorbacheva22@gmail.com (E.Y.G.); 2 Cell Biophysics Laboratory, SSC RF-IBMP RAS, 123007 Moscow, Russia; 3 Emergency and Extreme Medicine Department, FGBU DPO CGMA UDP RF, 121359 Moscow, Russia; wpx@mail.ru; 4 Medical and Biological Physics Department, I. M. Sechenov First Moscow State Medical University, 119991 Moscow, Russia

**Keywords:** cell motility, cytoskeleton, microfilament, tubulin, endometriosis

## Abstract

(1) Background: Endometriosis is a common pathology of the female reproductive system, often accompanied by pain and decreased fertility. However, its pathogenesis has not been sufficiently studied regarding the role of the cytoskeleton. In this study, we describe two clinical cases involving rare localization of extragenital endometriosis (umbilicus) and compare them with genital endometriosis of different localization (ovaries and uterus), as well as eutopic endometrium obtained with separate diagnostic curettage without confirmed pathology. (2) Methods: The relative content of actin and tubulin cytoskeleton proteins was determined by Western blotting, and the expression of genes encoding these proteins was determined by RT-PCR in the obtained intraoperative biopsies. The content of 5hmC was estimated by dot blot experiments, and the methylase/demethylase and acetylase/deacetylase contents were determined. (3) Results: The obtained results indicate that the content of the actin-binding protein alpha-actinin1 significantly increased (*p* < 0.05) in the groups with endometriosis, and this increase was most pronounced in patients with umbilical endometriosis. In addition, both the mRNA content of the ACTN1 gene and 5hmC content increased. It can be assumed that the increase in 5hmC is associated with a decrease in the TET3 demethylase content. Moreover, in the groups with extragenital endometriosis, alpha- and beta-tubulin content was decreased (*p* < 0.05) compared to the control levels. (4) Conclusions: In analyzing the results, further distance of ectopic endometrial foci from the eutopic localization may be associated with an increase in the content of alpha-actinin1, probably due to an increase in the expression of its gene and an increase in migration potential. In this case, a favorable prognosis can be explained by a decrease in tubulin content and, consequently, a decrease in the rate of cell division.

## 1. Introduction

In recent years, endometriosis has become an increasingly common gynecological pathology; one possible cause is the influence of various environmental factors on the onset and development of this disease. Endometriosis is the formation of foci of tissue that is similar to endometrial epithelium outside the uterine cavity. This pathogenesis contradicts one of the key paradigms of embryonic development, where each type of cell, after passing through all stages of differentiation and morphogenesis, remains in its microenvironment, interacting with neighboring cells and cell types. Accordingly, the formation of tissue foci in an uncharacteristic localization requires, on one hand, the opportunity for it to grow there, and on the other hand, changes in the tissue where it is embedded: there must be an opportunity for cell adhesion and immunological tolerance to the emerging “autotransplant”.

Recently, there is more and more evidence that the leading role in the adhesion of endometrial cells to the peritoneal mesothelium is played by the epithelial–mesenchymal transition which the mesothelium cells underwent [1]. Evidence of such a transition is a decrease in epithelial (E-cadherin, desmoplakin, mucin-1, occludin and claudin) and an increase in mesenchymal (N-cadherin, smooth muscle actin, vimentin, fibronectin, etc.) markers, which was observed in the defeat of the peritoneum and ovaries [2,3]. In addition, multidirectional changes in the activity of matrix metalloproteinases, which are observed in endometriosis [4], can lead to changes in the extracellular matrix, which promotes the attachment of endometrial cells with increased adhesion to various components of the extracellular matrix, including type IV collagen, laminin, vitronectin and fibronectin [5]. Further, after adhesion and invasion, the proliferation of endometrial cells and the growth of the ectopic focus will largely depend on angiogenesis, which is mediated by cytokines, leading primarily to an increase in the content of the vascular endothelial growth factor VEGF [6,7,8], and its levels correlate with the severity of the disease [9]. Therefore, VEGF can be considered as a potential target for the treatment of endometriosis. It has been shown that the use of oxytocin leads to a decrease in its level and correlates with a decrease in ectopic foci of endometriosis in a rat model [10].

At the same time, the adhesion of endometriumlike cells leads to the activation of macrophages and dendritic cells, the subsequent development of the immune response and the formation of immunological tolerance.

In women diagnosed with endometriosis, significantly higher concentrations of macrophages were found [11]. Moreover, in this case, peritoneal macrophages have an increased ability to secrete chemotactic protein-1 of monocytes-MCP-1 [12], which may indicate an increased recruitment of macrophages and be one of the main points in the pathogenesis of endometriosis [13]. Evidence in favor of this is the data obtained in the abovementioned work using rats as a model object [10]. The authors showed that oxytocin not only lowers the level of VEGF, but also reduces proinflammatory activation, decreasing the level of MCP-1 and TNF-α [10]. Macrophages carry out their protective function by phagocytosis, the secretion of cytokines and participation in the complement system. It has been shown that in the peritoneal fluid of women with endometriosis, the concentration of proteins of the complement system is higher than in the control [14,15]. At the same time, in the ectopic endometrioid foci localized on the peritoneum, and in the surrounding mesothelial tissue, there is a significant increase in immature dendritic cells that are unable to present the antigen, compared with the endometrium localized in the uterus, and with the mesothelial tissue, which is located much further from the site lesions [16], which may be one of the reasons for the development of immunological tolerance.

On the other hand, the formation of the ectopic foci requires the opportunity for ectopic tissue to migrate to the place of new localization, which could be connected with cytoskeleton dysregulation.

The most common localization of endometrial foci is the organs of the reproductive system: the uterus and ovaries. Adenomyosis is characterized by the presence of the benign proliferation of the endometrial glands and endometrial stroma in the myometrium. Retrospective studies show that the onset of the disease usually occurs between the ages of 30 and 40 and often coexists with leiomyoma; although menorrhagia is the most common symptom, approximately one-third of cases are asymptomatic [17]. Localization of the endometrioid cells in the ovary is the most common among ectopic foci; it is quite well understood and was first described in 1925 by Sampson, who theorized that retrograde menstruation was the cause of endometriosis [18]. It should be noted that the widely used marker of ovarian cancer, CA-125, may remain within the normal range, which probably depends on the degree of tumor differentiation [19,20].

The formation of endometrial foci in the abdominal wall is most often associated with previous gynecological interventions. Thus, it is believed that endometriosis of the abdominal wall is a rare disease that occurs after cesarean section or pelvic surgery and has a frequency of 0.03–1.5% in women with a similar history [21]. However, in this case, the ectopic focus is more often localized in the mesothelium of the abdominal wall and not on its outer side.

The rarest cases of endometrial foci formation outside the pelvic organs are cases of thoracic endometriosis. These cases are characterized by catamenial pleural pain, shortness of breath, and hemoptysis. Flieder et al. [22] described the clinical and pathological features of nine cases of pleuropulmonary endometriosis and the first case of pulmonary ectopic deciduosis. Pancytokeratin, estrogen and progesterone receptors were found on the foci of glandular epithelium. In this case, stromal cells were stained for estrogen and progesterone receptors as well as vimentin, actin, and desmin [22]—one of the key cytoskeletal proteins—which correlates with the leading role of the cytoskeleton in the migration, adhesion, invasion and proliferation of cells, including epithelial and stromal cells of the endometrium. Nevertheless, experimental data on the participation of the cytoskeleton in the pathogenesis of endometriosis are very few. A screening study aimed at identifying differentially expressed genes in patients with endometriosis shows that most of them encode proteins involved in the formation of focal-adhesive complexes, actin cytoskeleton, and in the signaling pathway of mitogen-activated phosphokinases [23].

It has been convincingly shown that estrogens and selective modulators of their receptors through extranuclear signaling cascades (G-proteins and Rho-associated kinases) regulate remodeling of the actin cytoskeleton, changing the adhesion and invasive ability of endometrial cells; 17-beta-estradiol has a specific effect [24]. The migration of endometrial stromal cells in response to 17-beta-estradiol and progesterone also changed in patients with endometriosis compared with the control group: 17-beta-estradiol stimulated the migration of endometrial stromal cells in the control and exhibited a stronger effect in the group with endometriosis, while progesterone only stimulated the migration of stromal cells in the endometriosis group [25].

An alternative hypothesis was proposed in [1], where the authors, after conducting a screening study, examined four genes (*WNT4*, *CDC42*, *ID4*, and *VEZT*) that control the actin cytoskeleton; it was suggested that the disruption of mesothelial barrier integrity as a result of aberrant gene expression may be a cause of the development of endometriosis. However, these genes are widely expressed in various cells and, along with mesothelium cells, can be dysregulated in endometrial cells. The latter can lead to a change in the content of proteins of the cytoskeleton, causing a high migration ability of endometrial cells in patients with endometriosis of various locations.

Since a necessary condition for cell motility and division is the interaction of microfilaments (formed by isoforms of actin and actin-binding proteins) and microtubules (formed by dimers of alpha- and beta-tubulin), the aim of the study was to determine the content of beta- and gamma-actin, alpha-actinin1 and 4, alpha- and beta-tubulin and expression of the encoding genes. In addition, we determined some epigenetic regulators of transcription and compared these molecular biological parameters with the clinical severity of the described cases.

## 2. Materials and Methods

### 2.1. Experimental Design

We describe 2 cases of rare extragenital localization of the endometrium in the umbilicus. Since these patients were admitted to the hospital at different times, for a correct comparison of molecular parameters and in order to avoid artifacts associated with different sample preparation, the biomaterial of other study groups was selected within 3 days in compliance with strict requirements for inclusion in a study group.

Five study groups were formed for comparison with the data of patients with umbilical endometriosis:

- Group 1 (*n* = 6): a control group of women with confirmed menopause for at least 3 years prior to the study who had no anamnesis of endometriosis. Biomaterial: eutopic endometrium without pathology, obtained by separate diagnostic curettage for a polyp (however, polyp tissue was not included in the tissues for investigation for all groups).
- Group 2 (*n* = 6): a control group of fertile women whose average age corresponded to the age of patients with endometriosis, but who had no anamnesis of endometriosis. Biomaterial: eutopic endometrium without pathology, obtained by separate diagnostic curettage for polyps.
- Group 3 (*n* = 5): a group of patients with adenomyosis. Biomaterial: eutopic endometrium, obtained by separate diagnostic curettage for polyps or menorrhagia.
- Group 4 (*n* = 4): a group of patients with external genital endometriosis. Biomaterial: ectopic endometrium (localization-ovaries and/or projection of the posterior surface of the cervix), obtained laparoscopically.
- Group 5 (*n* = 4): a group of patients with extragenital endometriosis. Biomaterial: ectopic endometrium (localization-pelvic peritoneum).
- Group 6 (*n* = 2): two patients with histologically confirmed umbilical endometriosis.

Immediately after preparation, the biomaterial was frozen in liquid nitrogen for subsequent isolation of protein and mRNA. Samples from all study groups, including a rare case, were processed in the same way and at the same time.

All patients were treated at Gynecology Department Clinical Hospital #1 (Volynskaya, Moscow, Russia). Examination of patients, presurgery preparation, surgery and management in the postsurgery period was carried out in accordance with the Order of the Ministry of Health of the Russian Federation of 1 November, 2012 N 572n “On approval of the procedure for the provision of medical care in the profile” obstetrics and gynecology (except for the use of assisted reproductive technologies) (with changes and additions on 17 January 2014, 11 June 2015, and 12 January 2016).

All patients were in stable clinical condition with no clinical, microbiological, or laboratory evidence of infection, encephalopathy, renal failure, or comorbidities including heart failure, pulmonary disease, malignancy, or diabetes mellitus. Written informed consent was obtained from each patient prior to participation in the study. The study design and procedures were approved by the Biomedicine Ethics Committee of the Institute of Biomedical Problems, Russian Academy of Sciences (Physiology Section of the Russian Bioethics Committee, Russian Federation National Commission for UNESCO, Permit #523/MSK/09/26/19) and conformed to the Declaration of Helsinki.

### 2.2. Protein Extraction and Western Blotting

Frozen tissues were used for protein extraction. Samples were homogenized in Laemmli buffer containing a protease inhibitor cocktail (Calbiochem, San Diego, CA, USA) on ice. After undergoing denaturing electrophoresis on polyacrylamide gels, proteins were transferred onto nitrocellulose membranes followed by staining with specific primary antibodies (Table 1), secondary antibodies and HRP-conjugated streptavidin-peroxidase. Membranes were developed using ECL substrate (Bio-Rad, Hercules, CA, USA). Signals were detected using a ChemiDoc XRS+ imaging system (Bio-Rad, Hercules, CA, USA) and analyzed using Image Lab Software (Bio-Rad, Hercules, CA, USA).

### 2.3. Evaluation of the Relative mRNA Level by Quantitative PCR

Total RNA from frozen tissues was isolated using an RNeasy Micro Kit (Qiagen, Hilden, Germany, #74004) according to the manufacturer’s instructions. Reverse transcription was performed using d(T)_15_ as a primer with 500 ng of RNA. qPCR was performed using the Mx300P system (Stratagene, La Jolla, CA, USA) using SYBR green with specific primers (Table 2). The expression of target genes was normalized to GAPDH and quantified by the 2^−ΔΔCT^ method.

### 2.4. Determination of 5-hydroxymethylcytosine (5hmC) Content in DNA by the Dot Blot Method

To determine methylation levels, total DNA was isolated from the frozen tissues using a DNA extraction kit (Syntol, Russia) based on the phenol/chloroform method according to the manufacturer’s instructions. The isolated DNA was applied to a nitrocellulose membrane for the preliminary measurement of concentrations and for denaturing (+95 °C for 5 min and then +4 °C for 3 min) at three dilutions—1 μg, 500 ng, and 200 ng. The membranes were air-dried, fixed to the membrane using ultraviolet light and incubated in 4% skim milk overnight at +4 °C. To evaluate the 5hmC content, we used specific primary antibodies (Abcam, UK, #ab214728, 1 μg/mL) and biotinylated goat anti-rabbit IgG antibodies (Jackson ImmunoResearch Lab., Inc., West Grove, PA, USA, 1:10,000) as secondary antibodies. Then, the membranes were treated with a streptavidin solution that was conjugated with horseradish peroxidase (Sigma, Darmstadt, Germany) at a dilution of 1:10,000. Dots were detected using 3,3′-diaminobenzidine (Merck, St. Louis, MA, USA). ImageJ (https://imagej.net/Fiji, access date: 2 September 2020) was used to analyze the data.

### 2.5. Statistical Analysis

Statistical analysis was performed using GraphPad Prism 7.03 software. Each experiment was performed at least three times and yielded consistent results. The Mann–Whitney U-test was used to assess differences between groups. Data were presented as median ± range. A two-tailed value of *p* < 0.05 was considered statistically significant.

All the methods were carried out in accordance with the relevant guidelines and regulations.

## 3. Results

### 3.1. Clinical Observation

The anamnesis of all patients was similar—for all of them it was a first abdominal surgery. There were no differences in typical clinical characteristics (Table 3): hemoglobin level and leukocytes count were in the limits of the normal values. Each study group consisted of patients with only the focus of endometriosis that belongs to this group. In group 3, adenomyosis was the only pathological process. In group 4, the patient had only one ovarian cyst without signs of adenomyosis or extragenital endometriosis. Group 5 included patients with 1–3 lesions on the peritoneal mesothelium, without signs of genital endometriosis or adenomyosis. Patients with umbilical endometriosis had foci of ectopic endometrium only in the navel (there were no signs of adenomyosis, genital endometriosis and foci on the peritoneum).

**Figure 1:**
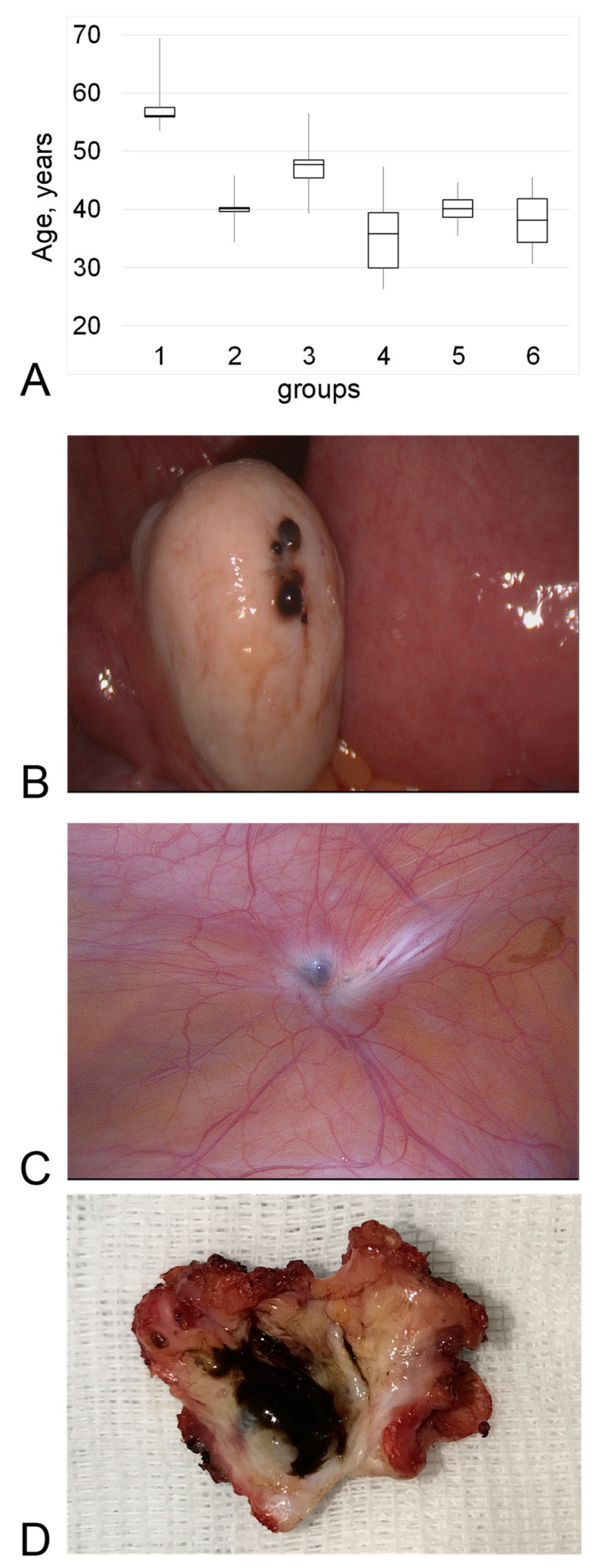
Comparison of groups. (**A**)—Average age. (**B**)—Typical intrasurgery picture of the endometrioid ovarian cyst (group 4—genital endometriosis). (**C**)—Typical intrasurgery picture of the ectopic endometrium localized on the mesothelium (group 5—extragenital endometriosis). (**D**)—postsurgery picture of the umbilicus with a focus of ectopic endometrium. All pictures were taken by Konstantin A. Toniyan during surgery with the informed consent of the patient.

The mean age of the patients in the postmenopausal control group was, as expected, higher than that of the patients in the other groups (Figure 1A). The average age of the patients in the remaining groups did not differ from each other.

The severity of the pathological process correlated with its prevalence. Patients in group 1 and group 2 had no complaints—the polyp was an accidental finding during a routine gynecological examination. The sizes of the polyp between the groups did not differ significantly. The thickness of the endometrium in group 2 was consistently higher than in group 1 (postmenopausal) and corresponded to the day of the menstrual cycle.

Patients with adenomyosis (group 3) presented with algomenorrhea and metrorrhagia as the main complaints. A single polyp was found in 4 out of 5 patients and had dimensions similar to those for group 1 and group 2. However, the thickness of the endometrium in this group was significantly higher by 67% (*p* < 0.05) compared with group 2, and the structure was heterogeneous.

Patients with genital endometriosis (group 4) (Figure 1B), had menstrual irregularities in addition to algomenorrhea. The size of the ovarian cyst was 39 ± 4 mm in diameter.

In the group with extragenital endometriosis (group 5), the localization of endometrial foci on the mesothelium was noted most often, and mesothelium was visually characterized with a local inflammatory process (Figure 1C). The patients in this group, in addition to the complaints characteristic of the previous groups, had severe pelvic pain, which significantly reduced their quality of life. The size of the foci of the ectopic endometrium was 2–3 mm in diameter (assessed during surgery). Patients in the group had from 2 to 4 loci on the mesothelium.

Two patients with umbilical endometriosis, who we separated into a distinct group (group 6) due to the exceptional prevalence of the process, noted, first of all, a pronounced pain syndrome in the area of localization of the endometriotic infiltrate. In one case (Figure 1D), the excised navel had one large lesion (10 mm) of the ectopic endometrium (in an younger patient), and the second patient had multiple (histologically observed—5) small lesions (about 0.8 mm), that merged into one.

The mean age of the patients in the postmenopausal control group was, as expected, higher than that of the patients in the other groups (Figure 1A). The average age of the patients in the remaining groups did not differ from each other. The anamnesis of all patients was similar.

The severity of the pathological process correlated with its prevalence. Patients with adenomyosis (group 3) presented with algomenorrhea and metrorrhagia as the main complaints. Patients with genital endometriosis (group 4), mainly with endometrioid ovarian cysts (Figure 1B), had menstrual irregularities in addition to algomenorrhea. In the group with extragenital endometriosis (group 5), the localization of endometrial foci on the mesothelium was noted most often, which was characteristic of a local inflammatory process (Figure 1C). The patients in this group, in addition to the complaints characteristic of the previous groups, had severe pelvic pain, which significantly reduced their quality of life. Two patients with umbilical endometriosis, who we separated into a distinct group (group 6) due to the exceptional prevalence of the process, noted, first of all, a pronounced pain syndrome in the area of localization of the endometriotic infiltrate. In one case (Figure 1D), the excised navel had one large lesion of the ectopic endometrium (in a younger patient), and the second patient had multiple small lesions.

### 3.2. Cytoskeletal Proteins ant Its mRNA Content

**Figure 2:**
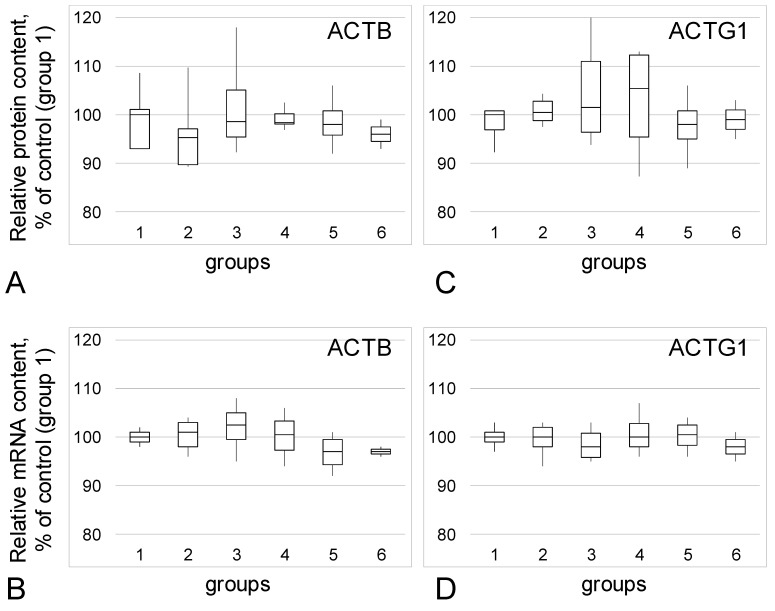
Relative content of actin isoforms and its mRNA. (**A**)—Beta-actin (ACTB) protein. (**B**)—Beta-actin mRNA. (**C**)—Gamma-actin (ACTG1) protein. (**D**)—Gamma-actin mRNA. 1—Group 1: Postmenopausal patients without endometriosis in their anamnesis. 2—Group 2: Patients without endometriosis in their anamnesis of average age similar to women from groups with different type of endometriosis. 3—Group 3: Patients with adenomyosis. 4—Group 4: Patients with genital endometriosis. 5—Group 5: Patients with extragenital endometriosis. 6—Group 6: Two patients with umbilicus endometriosis.

The relative content of beta-actin protein (Figure 2A) and mRNA (Figure 2B), as well as gamma-actin (Figure 2C,D), did not change between all studied groups.

**Figure 3:**
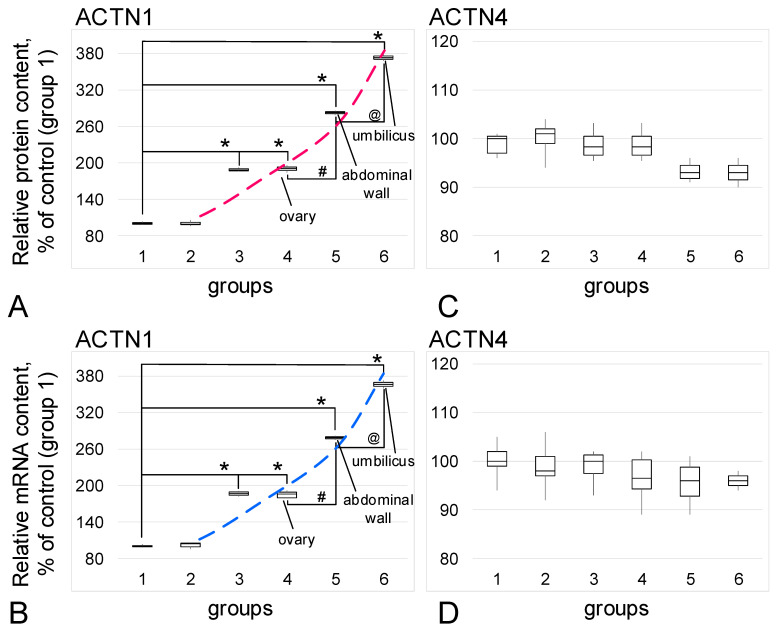
Relative content of alpha-actinin isoforms and their mRNA. (**A**)—Alpha-actinin1 (ACTN1) protein. (**B**)—Alpha-actinin1 mRNA. (**C**)—Alpha-actinin4 (ACTN4) protein. (**D**)—Alpha-actinin4 mRNA. *—*p* < 0.05 in comparison with control group 1 (women in postmenopause without endometriosis in their anamnesis). #—*p* < 0.05 in comparison with group 4 (women with genital endometriosis). @—*p*< 0.05 in comparison with group 5 (women with extragenital endometriosis, but localized in the small pelvis). Red and blue dashed lines reflect trends in protein and mRNA content, respectively. Group designations are the same as above.

The relative content of the isoform of actin-binding protein alpha-actin1 (Figure 3A) and its mRNA (Figure 3B) increased in the groups with confirmed endometriosis. An increase in the prevalence of endometriosis correlated with an increase in the content of ACTN1: in the group with adenomyosis (group 3) the content of protein and mRNA increased by 89% and 87% (*p* < 0.05), respectively, while in the group with genital endometriosis (group 4), there were increases of 91% and 86% (*p* < 0.05). For the group with extragenital endometriosis, predominantly with the involvement of the pelvic peritoneum (group 5), the increases were 183% and 179% (*p* < 0.05), and in cases with umbilical endometriosis (group 6), the increases were 273% and 266% (*p* < 0.05), respectively. Moreover, in group 5, the content was significantly higher (*p* < 0.05) than in groups 3 and 4, and in group 6, it was significantly higher than in group 5 (*p* < 0.05).

For another alpha-actinin isoform, alpha-actinin4, no changes in the relative protein content (Figure 3C) or mRNA (Figure 3D) were observed.

**Figure 4:**
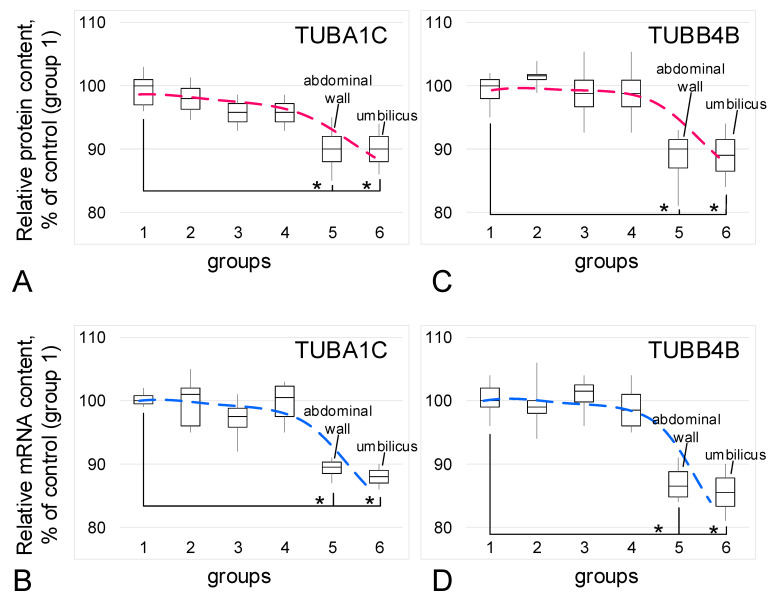
Relative content of tubulin isoforms and its mRNA. (**A**)—Alpha-tubulin (TUBA1C) protein. (**B**)—alpha-tubulin mRNA. (**C**)—Beta-tubulin (TUBB4B) protein. (**D**)—Beta-tubulin mRNA. *—*p* < 0.05 in comparison with control group 1 (women in postmenopause without endometriosis in their anamnesis). Red and blue dashed lines reflect trends in protein and mRNA content, respectively. Group designations are the same as above.

The relative content of the main structural components of microtubules, tubulin isoforms (protein and mRNA), did not change between the control groups and the groups with adenomyosis (group 3) or genital endometriosis (group 4). In the groups with extragenital endometriosis (group 5) and umbilical endometriosis (group 6), a decrease of 10–12% in the relative content of protein and mRNA of alpha-tubulin (Figure 4A,B) and beta-tubulin (Figure 4C,D) was observed (*p* < 0.05) in each case.

### 3.3. Acetylase/Deacetylase Relative Content

**Figure 5:**
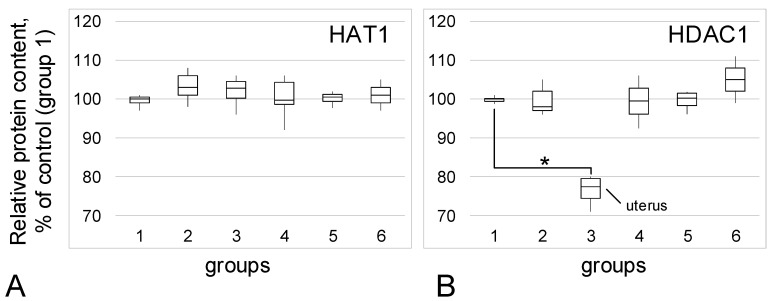
Acetylase/deacetylase relative content. (**A**)—Histone acetylase HAT1 relative content. (**B**)—Histone deacetylase HDAC1 relative content. *—*p* < 0.05 in comparison with control group 1 (women in postmenopause without endometriosis in their anamnesis). Group designations are the same as above.

The content of histone acetylase HAT1 did not change among the study groups (Figure 5A). The content of HDAC1 histone deacetylase (Figure 5B) also practically did not change between the control groups and groups 4, 5, and 6. However, in group 3 (patients with adenomyosis), the relative content of HDAC1 was 23% lower than it was in the control (*p* < 0.05).

### 3.4. 5hmC Content and Methylase/Demethylase Relative Content

**Figure 6:**
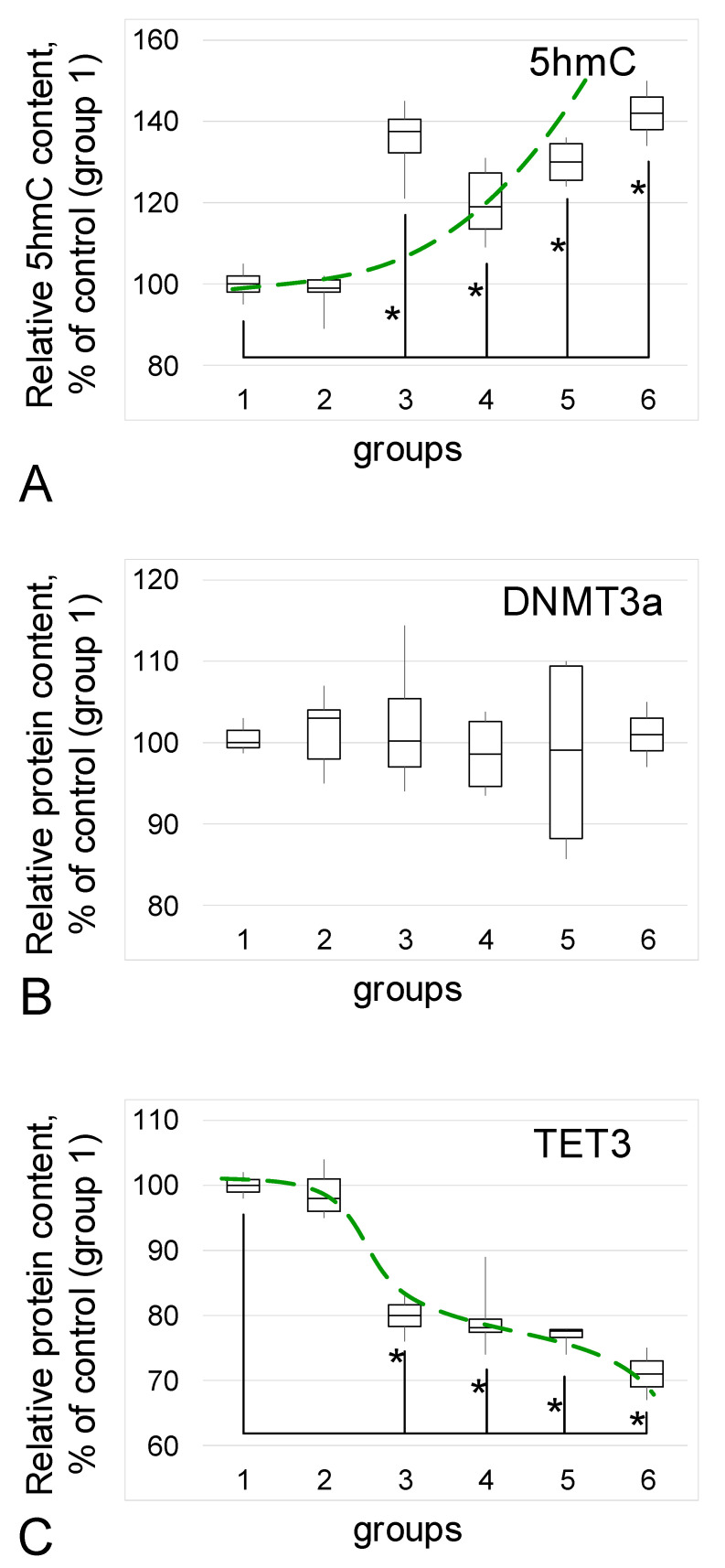
Relative content of 5-hydroxymethylcytosine (5hmC) and methylase/demethylase proteins. (**A**)—5hmC. (**B**)—Methylase DNMT3a relative content. (**C**)—Demethylase TET3 relative content. *—*p* < 0.05 in comparison with control group 1 (women in postmenopause without endometriosis in their anamnesis). Green dash line reflects trends. Group designations are the same as above.

The content of the methylation intermediate product, 5-hydroxymethylcytosine (5hmC), increased in groups with confirmed endometriosis (Figure 6A): in group 3 the increase was 38% (*p* < 0.05), in group 4, it was 19% (*p* < 0.05), in group 5 it was 30% (*p* < 0.05), and in group 6 it was 42% (*p* < 0.05).

The DNMT3a methylase content did not change in any of the study groups compared to control group 1 (Figure 6B).

The content of active demethylase of the ten-eleven translocation protein TET3 (Figure 6C) decreased in groups with endometriosis: in group 3 the decrease was 20% (*p* < 0.05), in group 4 it was 22% (*p* < 0.05), in group 5 it was 23% (*p* < 0.05), and in group 6 it was 29% (*p* < 0.05).

## 4. Discussion

Most of the clinical facts and numerous cell–molecular studies allow us to consider endometriosis as a hormone-dependent inflammatory disease, which is often associated with the activation of the peripheral and central nervous systems and the formation of pain syndrome as well as infertility [26,27,28]. Estrogen promotes the survival of endometrial cells in ectopic foci, the inflammatory response and disease progression [29,30], while aromatase inhibitors significantly reduce the severity of disease symptoms [31,32]. During menopause, there is a natural decrease in estrogen synthesis. Therefore, we chose postmenopausal women with no anamnesis of endometriosis as the main control group for the purposes of our study, which is primarily associated with the assessment of cytoskeletal factors that ensure cell migration.

The second control group consisted of women of reproductive age who, at the time of the study, also had no history of endometriosis. Despite the fact that the parameters studied in these two groups did not differ significantly, we noticed a more pronounced variability in the values, which may indicate the heterogeneity of the group. The size of the groups was limited due to the peculiarities of the studied parameters: to unify the results, all molecular studies were carried out simultaneously (the collection of biomaterials was carried out within 3 days from cases of umbilical endometriosis). Therefore, it can be assumed that an increase in the number of participants in this group might lead to its division: some members might possibly develop endometriosis, and some will not.

Despite a large number of studies, the pathogenesis of endometriosis remains poorly understood due to the heterogeneity of this disease. In particular, it is still not entirely clear what influences the prevalence of the process in different patients. In this study, we tested the hypothesis that in the case of a widespread process, the main cytoskeletal proteins that determine the ability of cells to migrate can be dysregulated.

Therefore, umbilical endometriosis was of particular interest. In general, the localization of lesions on the abdominal wall is a rather rare phenomenon—0.03–1% of all cases of endometriosis [33], but even in this statistic, the focus is, in most cases, localized on the inner (mesothelium) side. The formation of a primary focus in the umbilicus, which is also known as Villar’s nodule, in the absence of an anamnesis of any surgery of these patients, is extremely rare [34,35,36]. In an adult, the umbilicus is a scar—a dense connective tissue formation, consisting mainly of collagen. In other words, the formation of an ectopic locus of the endometrium on the outer side of the abdominal wall, in the umbilicus, implies the migration of cells through structurally different layers: peritoneal mesothelium, umbilical fascia, different ligaments, linea alba, etc. toward the umbilical ring. Accordingly, it is believed that these endometrial cells will overexpressed the cytoskeleton proteins required for such migration.

We did not observe changes in the content of actin isoforms in any of the study groups. However, the content of one of the actin-binding protein isoforms, alpha-actinin1, as well as its gene expression, increased in the groups with endometriosis; this increase was more pronounced in the case of umbilical endometriosis. Moreover, the content of alpha-actinin4, another isoform of this protein, did not change. It is well known that various forms of cancer are associated with an increase in alpha-actin isoforms, predominantly alpha-actinin4—see review [37]. There are few data for the association of alpha-actinin1 and cancer, but they also indicate an increase in the migration potential of various cells with ACTN1 overexpression, for example, in the case of esophageal squamous cell carcinoma [38], basal-like breast cancer [39], and myxofibrosarcoma [40].

Nevertheless, an increase in ACTN1 does not lead to malignancy of the process in endometriosis, which would be expected for forms of extragenital endometriosis due to high ACTN1 content. However, the data obtained indicate that the main protein content and gene expression of microtubules, alpha- and beta-tubulin, in these cases are reduced. Microtubules are directly involved in the division process, forming the division spindle and thus, are involved in tumor pathogenesis [41,42]. Reducing the formation of microtubules significantly improves the prognosis in cancer [43,44]. In addition to inhibiting polymerization, a decrease in the content of tubulin monomers can lead to a decrease in microtubule formation. Perhaps, in our case, a decrease in tubulin synthesis in the foci of extragenital endometriosis may determine the benign quality of the process. However, the question remains whether there are any associations in the observed cases with changes in the expression of genes encoding alpha-actinin1 and tubulin isoforms.

It has been demonstrated that the histone deacetylase inhibitors tubastatin A and suberoylanilide hydroxamic acid reduce the activity of histone acetylases and lead to a decrease in the content of alpha-tubulin [45]. In some cases of endometriosis, an increase in the activity of histone deacetylases is found [46,47,48]. We determined the content of acetylase HAT1 and deacetylase HDAC1 in biopsies of control groups and groups with endometriosis and found no differences. There was a decrease in the HDAC1 content in the group with adenomyosis, but there were no changes in the tubulin content in this group.

Therefore, to determine possible factors influencing the expression of cytoskeletal genes, we determined the content of the intermediate product of genome methylation (5-hydroxymethylcytosine) and the enzymes that regulate its content. In all groups with endometriosis, the 5hmC content was higher than the control level, while the active demethylase TET3 content was reduced and the methylase content remained unchanged. Active demethylases of the TET (ten–eleven translocation) family carry out protein-mediated oxidative catalysis of 5hmC in DNA with the formation of 5hmC [49,50]. It can be assumed that the accumulation of 5hmC in ectopic endometrial cells may be associated with a decrease in demethylase TET; in other words, the normal process of methylation/demethylation of the genome in this case is shifted toward the methylated state. In general, an increase in genome methylation is associated more often with a decrease in the expression level as a result of tighter binding of methylated DNA to the nucleosome [51,52]. Nevertheless, some genes can avoid this and be hypomethylated during general hypermethylation, both in various diseases and during adaptation to extreme conditions [53,54,55], demonstrating a high level of expression against the background of transcriptional silencing, which may explain the high level of expression of ACTN1 in the described cases of endometriosis. For example, for alpha-actinin1 in the tissues of the heart, lungs, and testes under conditions of weightlessness simulation, it was shown that CpG islands in the promoter region avoid global methylation and the expression of ACTN1 in these cases increases [54]. However, changes in the methylation level in introns of the ACTN1 gene, do not correlate with its expression level [56]. In general, mammals have a large number of possibilities for the regulation of gene expression, including RNA interference and various chromatin modifications, which cannot be excluded in the described cases, thus requiring further research. 

## 5. Conclusions

Thus, although endometriosis is a benign proliferative disease, it shares characteristics with tumor processes, in particular, high migration potential, inflammatory conditions, invasion of adjacent tissues, induction of angiogenesis, and resistance to apoptosis [57]. In summary, we can assume that the migration of endometrial cells and the formation of ectopic foci, especially in cases of extragenital localization and even the umbilicus, may be associated with an increase in the content and expression of alpha-actinin1, which is also characteristic of cancer. At the same time, reducing the tubulin content in these lesions can prevent malignancy. It should be noted that in the case of ovarian localization of endometrial foci, against the background of an increase in ACTN1 and a decrease in tubulin, was not observed, which may be an unfavorable prognostic sign. Malignant transformation of endometriosis is a well-documented, albeit rare, phenomenon that most often occurs in the ovaries [58,59].

In conclusion, we showed that in the foci of ectopic endometrium of any localization, the content of one of the actin-binding proteins, alpha-actinin1, increases, both at the protein level and at the mRNA level, but in the foci of extragenital localization, the content of tubulin decreases. At the same time, the content of an intermediate methylation product, 5hmC, increases against the background of a decrease in the content of active demethylase of the TET family. The most pronounced changes are observed in rare clinical cases—with umbilical localization.

The limitations of the study are primarily due to the fact that it is a descriptive study, in view of the study of human samples, which makes it impossible to directly test some of the assumptions. Moreover, in order to avoid artifacts and unify sample preparation, we collected material from all research groups within three days, making strict requirements for inclusion in one or another group. This led to a decrease in the study sample size, although the use of adequate statistical methods revealed the above-described significant differences. However, we believe that the results obtained may be of interest in the analysis of the migration potential of endometrial cells and the development of methods for its reduction in order to prevent the development of advanced endometriosis.

## Figures and Tables

**Table 1:** Primary antibodies.

Protein	Manufacturer with Catalog Number, Dilution
ACTB (beta-actin, 42 kDa)	Santa Cruz Biotechnology, Inc., Paso Robles, CA, USA, #sc-81178, 1:300
ACTG1 (gamma-actin, 42 kDa)	Santa Cruz Biotechnology, Inc., Paso Robles, CA, USA, #sc-65638, 1:100
ACTN1 (alpha-actinin1, 103 kDa)	Santa Cruz Biotechnology, Inc., Paso Robles, CA, USA, #sc-17829, 1:500
ACTN4 (alpha-actinin4, 102 kDa)	Santa Cruz Biotechnology, Inc., Paso Robles, CA, USA, #sc-393495, 1:100
TUBA1C (alpha-tubulin, 50 kDa)	Abcam, Cambridge, UK, #ab52866, 1:1000–1:50,000
TUBB4B (beta-tubulin, 50 kDa)	Abcam, Cambridge, UK, #ab179513, 1:1000
TET3 (tet methylcytosine dioxygenase 2, 179 kDa)	Abcam, Cambridge, UK, #ab139805, 2 mkg/mL
DNMT3A (120 kDa)	Abcam, Cambridge, UK, #ab2850, 2 mkg/mL
HAT1 (histone acetylase, 45 kDa)	Abcam, Cambridge, UK, #ab194296, 1:1000
HDAC1 (histone deacetylase, 55 kDa)	Abcam, Cambridge, UK, #ab109411, 1:1000

**Table 2:** Primer sequences and product sizes.

Gene	Primer Sequence, Forward/Reverse (5′… 3′)	Product Size, bp
ACTB	CTCGCCTTTGCCGATCC/TCTCCATGTCGTCCCAGTTG	298
ACTG1	GTTTCTCTGCCGGTCGCAAT/CCGACGATGGAAGGAAACA	126
ACTN1	GTGTCCGCCTAGTTCAGTGT/ATTGACCGCCAACACTTTGC	251
ACTN4	AATCCAATGAGCACCTCCGC/TGGTGTGCTTGTTGTCGAAG	243
TUBA1C	CCGGCCACCCTTTCACTACT/CTCATCGTCTCCTTCAGCACT	76
TUBB4B	CCACCTCGGGGGCTAAAAAT/CCTCGGTGAACTCCATCTCG	163

**Table 3:** The clinical and pathological parameters of the patients.

Parameter	Group 1 (Control)	Group 2 (Control)	Group 3 (Adenomyosis)	Group 4 (Genital EM)	Group 5 (Extragenital EM)	Group 6 (Umbilical EM)
Hemoglobin, g/L	139 ± 8	127 ± 5	123 ± 4	129 ± 7	136 ± 5	123 ± 0.5
Leukocytes, 10^9^/L	6.4 ± 0.6	6.6 ± 0.9	6.9 ± 1.4	6.3 ± 0.3	7.0 ± 0.5	7.0 ± 0.9
Cause of the surgery	Polyp in the uterus	Polyp in the uterus	Polyp in the uterus or menorrhagia	Ovarian cyst	Ectopic loci at the pelvic peritoneum	Ectopic loci in the umbilicus
Stage of the process (size of the neoplasm, mm)	9.3 ± 1.3	8.5 ± 0.9	11 ± 2	39 ± 4	2–3	10 (YP), 0.8 (OP)
Endometrial thickness, mm (Seventh day of the menstrual cycle, for group 1—any day)	5.0 ± 0.6	8.0 ± 0.7	13.3 ± 0.3 *	7.3 ± 0.5	7.5 ± 1.1	7.7 ± 0.1
Severity of the process (number of pathological loci)	1	1–2	1	1	2–4	1 (YP), 5 (OP)

* *p* < 0.05 in comparison with control group 2 (depends on the day of menstrual cycle). EM—endometriosis. YP—younger patient with umbilical EM. OP—older patient with umbilical EM. We did not compare size of the neoplasm between groups 1, 2 or 3 and 4 or 5, 6 due to principally different origin.

## Data Availability

All data generated or analyzed during this study are included in this article.
